# Diabetogenic and Obesogenic Effects of Cadmium in Db/Db Mice and Rats at a Clinically Relevant Level of Exposure

**DOI:** 10.3390/toxics10030107

**Published:** 2022-02-23

**Authors:** Jessica Nguyen, Arjun Patel, Andrew Gensburg, Rehman Bokhari, Peter Lamar, Joshua Edwards

**Affiliations:** 1 Chicago College of Pharmacy, Midwestern University, Downers Grove, IL 60515, USA; j10nnguyen@gmail.com; 2 Arizona College of Osteopathic Medicine, Midwestern University, Glendale, AZ 85308, USA; arjunbapa24@gmail.com; 3 Chicago College of Osteopathic Medicine, Midwestern University, Downers Grove, IL 60515, USA; andrew.gensburg@midwestern.edu (A.G.); rehman.bokhari@midwestern.edu (R.B.); 4 College of Graduate Studies, Midwestern University, Downers Grove, IL 60515, USA; plamar@midwestern.edu

**Keywords:** cadmium, leptin, db/db mouse, diabetes mellitus, obesity

## Abstract

Studies show an association between cadmium (Cd) exposure and prediabetes or type II diabetes mellitus. We have previously reported that Cd causes decreased levels of serum leptin in rats following 12 weeks of daily Cd dosing (0.6 mg/kg/b.w./day). Since leptin plays an important role in metabolism, we examined the effects of Cd on rats and db/db mice, which are deficient in leptin receptor activity. We gave rats and mice daily subcutaneous injections of saline (control) or CdCl_2_ at a dose of 0.6 mg/kg of Cd for 2 weeks, followed by 2 weeks of no dosing. At the end of the 4-week study, exposure to Cd resulted in a more rapid increase in blood glucose levels following an oral glucose tolerance test in db/db vs. lean mice. During the two weeks of no Cd dosing, individual rat bodyweight gain was greater (*p* ≤ 0.05) in Cd-treated animals. At this time point, the combined epididymal and retroperitoneal fat pad weight was significantly greater (*p* ≤ 0.05) in the Cd-treated lean mice compared to saline-treated controls. Although this pilot study had relatively low N values (4 per treatment group for mice and 6 for rats) the results show that clinically relevant levels of Cd exposure resulted in diabetogenic as well as obesogenic effects.

## 1. Introduction

Cadmium (Cd) is a ubiquitous environmental contaminant that is a group 1 carcinogen with toxic effects in lung, liver, testicular, kidney and bone tissues [1]. The kidney is considered the primary target organ of Cd toxicity with concentrations reaching the highest levels in the renal cortex over time. Human Cd content ranges widely in the kidney cortex from approximately 25 to 84 ug/g of wet tissue weight in individuals with non-occupational exposure [2,3]. Differences in geographical location, gender and shifting trends in tobacco use are potential causes for the wide variation. Regardless, several studies show that Cd accumulates in the overall kidney (cortex and medulla) over time and a plateauing effect with peak concentrations occurs after roughly 50 years of age; for a review, see [4].

While Cd is primarily considered a nephrotoxicant, there is a body of literature showing significant correlations between exposure to Cd and the prevalence of prediabetes and/or type II diabetes mellitus [5,6,7]. However, it should be noted that not all studies have found significant associations between markers of Cd exposure and diabetes mellitus [8]. Numerous short-term and long-term in vivo Cd exposure models have shown Cd to cause hyperglycemia and disrupt glucose homeostasis in experimental animals [9,10,11]. The exact mechanism of action of the Cd-induced disruption of glucose homeostasis is unknown. However, overall pancreatic β cell dysfunction and specifically impaired glucose-stimulated insulin release are likely factors. It should be noted that type I diabetes is characterized by abrupt and complete β cell death or dysfunction, while type II diabetic patients may slowly develop β cell dysfunction over several years or decades [12]. Cd accumulates within human pancreatic β cells and alters glucose-stimulated insulin release in vitro [13]. In addition, in individuals with occupational exposure to Cd, serum insulin, from 10 to 12 h fasted individuals, is significantly lower in individuals with elevated urinary Cd levels [14]. Additional mechanisms by which Cd may alter glucose homeostasis involve changes in glucose transporter expression in adipocytes [15] and increases in renal and hepatic gluconeogenesis [16,17]. Taken together, this would indicate that Cd likely has multiple effects in multiple tissues to cause alterations in glucose homeostasis.

The relationship between Cd exposure and obesity is not as well studied and appears to be complex. One report shows a negative correlation between blood Cd levels and body mass index values in a population in China [18]. Studies such as these become difficult to interpret when considering that cigarette smoke is the primary source of non-occupational Cd exposure [19] and compounds found in cigarette smoke, namely nicotine, are responsible for weight loss [20]. The effects of tobacco use are so great that fear of post-cessation weight gain is a major reason why smokers fail to initiate smoking cessation efforts [20].

This study was primarily intended to examine potential diabetogenic effects of Cd in db/db mice; a mutant strain that expresses a non-functional ob-Rb subtype of the leptin receptor [21]. Leptin is a neuroendocrine hormone with white adipose tissue being a primary source. Expression of leptin receptors occurs in the brain and other organs and impacts satiety, thermoregulation and even sexual behavior; for a review, see [22]. Db/db mice are a well characterized model of type II diabetes that show diminished islet β-cell number, insulin resistance and the onset of obesity at 3–4 weeks of age [23]. In addition, Sprague Dawley rats were examined because many Cd toxicological studies use this species as an animal model of both acute and chronic intoxication.

## 2. Materials and Methods

### 2.1. Animals

Young adult male Sprague Dawley rats (initial bodyweight was 283 ± 1.4 g, mean ± SE), male db/db mice (BKS.Cg- + Lepr^db^/+ Lepr^db^/OlaHsd) (initial bodyweight was 29.1 ± 0.5 g, mean ± SE) and male “lean” control mice (BKS.Cg-Dock7^m^ +/+ Lepr^db^/OlaHsd) (initial bodyweight was 22.1 ± 0.3 g, mean ± SE) were purchased from Harlan Laboratories Indianapolis, IN. All animal studies were reviewed and approved by the Institutional Animal Care and Use Committee at Midwestern University, Downers Grove, IL; approved IACUC file # 1963; original approval date: April 2013. Animals were housed in an AAALAC accredited facility with 14:10 h light/dark cycle and unfettered access to standard rodent chow and water, except that food was withheld 5 h prior to and during the oral glucose tolerance test (OGTT).

### 2.2. Cd Dosing

Animals were randomly assigned to treatment groups; Cd-treated vs. saline control. Rats (N = 6), db/db mice (N = 4) and lean mice (N = 4) assigned to the Cd treatment group were given daily subcutaneous injections of Cd at the dose of 0.6 mg/kg of Cd in the form of CdCl_2_ for 5 days a week for two weeks (10 doses in total) followed by two weeks of non-Cd dosing. Rats (N = 6), db/db mice (N = 4) and lean mice (N = 4) assigned to the saline control treatment group received parallel daily injections (10 total) of equal volume of vehicle alone (0.9% sodium chloride Sigma cat. # S8776, Sigma-Aldricht, St. Louis, MO, USA) followed by two weeks of non-saline dosing. The two weeks of subsequent non-Cd dosing allows for the biotransformation of Cd to occur (i.e., Cd conjugates to form and become deposited in tissues such as the renal cortex) so all potential forms of Cd would be present and all potential Cd effects could take place in the animals [24].

### 2.3. Oral Glucose Tolerance Test (OGTT)

Animals were fasted for 5 h before a single oral dose of glucose solution (2 g/kg) was administered using an appropriately sized gavage needle. Immediately prior to the oral glucose dosing, animal fasting blood glucose was determined by an Equaline^®^ Brand (Ft. Lauderdale, FL, USA) portable glucose meter. Blood samples were collected by nicking the tip of the tail with a 26-gauge syringe needle. For all samples, care was taken to collect blood within a 2 min time period after the animal was touched, so that the handling of the animal would not artificially increase the blood glucose value.

### 2.4. Body and Fat Pad Weight

Rats were weighed once a week and mice were weighed twice a week at regular intervals on electronic balances. At the end of the study, the left and right epididymal and retroperitoneal fat pads for each animal were collected and then weighed using an electronic balance.

### 2.5. Determination of Metabolic Indicators in Serum

The abdominal cavities of animals were opened after they were anesthetized with ketamine 67 mg/kg and xylazine 7 mg/kg (i.p.). Pancreata were removed for later histological analyses and blood samples were collected from the exposed vena cava using syringes and allowed to clot at room temperature for 30 min. The blood samples were then centrifuged at 2000× *g* for 15 min at 4 °C. The resulting top layer of clear serum was carefully pipetted (50–100 µL) to new labelled microcentrifuge tubes while avoiding any clotted blood and stored at −80 °C until analyzed. Leptin, insulin and glucose-dependent insulinotropic polypeptide (GIP) levels were quantified using commercially available ELISA kits using the manufacturer’s recommended protocol (Crystal Chem Inc., Downers Grove, IL, USA)—mouse leptin cat. # 90030, rat insulin cat. # 90060, mouse insulin cat. # 90080, rat GIP cat. # 81,516 and mouse GIP cat. # 81517. GIP is a gastrointestinal hormone that is secreted from enteroendocrine K cells and pancreatic alpha cells and acts to potentiate insulin release [25]. Absorbance readings at 450 nm of the chromogenic substrate, 3,3′,5,5′-Tetramethylbenzidine (TMB), were recorded on a Beckman Coulter (Indianapolis, IN, USA) model DTX 880 multimode plate reader. Absorbance values from unknown serum samples were quantified from known standards supplied by the manufacturer of the ELISA kit using non-linear regression analysis of the standard curves. Only standard curves with r^2^ values of 0.95 or greater were used to quantify serum samples with the absorbance values of unknown samples with the range of known standards.

### 2.6. Morphometric Analyses of Pancretic Islets

Pancreata were removed immediately following the collection of blood. The tissues were immediately placed in 10% formalin solution, stored in vials, embedded in paraffin and sectioned at 5 µm onto glass microscope slides (AML Labs, St. Augustine, FL, USA). Sectioned tissue underwent deparaffinization in xylene for two minutes and rehydrated in a series of washes in graded ethanol solutions (100%, 95%, 70%) for two minutes each, followed by two minutes in tap water. Tissue sections were incubated hematoxylin for 30 min, rinsed three times in running tap water then incubated in 1% HCl in 70% ethanol for 30 s, then a further 30 s in 0.5% lithium carbonate, then incubated with eosin-phloxine for 3 min. Sections were then dehydrated in a series of alcohols (95%, 95%, 100% and 100%) for two minutes each. After two changes of two minutes each in xylene, sections were mounted under glass coverslips with Permount (Fisher Scientific, cat. # SP15-500). Images were captured using a high-resolution Evolution MP Color digital camera (Media Cybernetics, Rockville, MD, USA) on a Nikon Eclipse E400 epifluorescent microscope (Melville, NY, USA). Researchers blind to the treatment effect or groups analyzed various quantitative aspects of stained islets including area, diameter (minimum), diameter (maximum), diameter (mean), perimeter, roundness, cell count and cell number per islet surface area utilizing the image capture and analysis software Image-Pro Plus (v. 7) (Media Cybernetics, Rockville, MD, USA). Ten images containing one or two islets were examined per animal per treatment group.

### 2.7. Determination of Cd Content in the Renal Cortex

Whole kidneys were removed from the opened abdominal cavity at the end of the experiment. The renal capsule was removed and the kidney cut in cross sections (transverse plane). The renal cortex was then identified from the tissue slices and removed whilst being careful to not include any of the renal medulla. Renal cortex samples were then stored at −80 °C in microcentrifuge tubes until the metal content was determined. Metal content was determined for all samples by the Michigan State University Veterinary Diagnostic Laboratory (East Lansing, MI, USA) using inductively coupled plasma mass spectrometry. This is an American Association of Veterinary Laboratory Diagnosticians (AAVLD) accredited laboratory and performs all tests in compliance with the standards set forth by the accrediting body. Briefly, 0.2 g of tissue was digested in Teflon containers with 2 mL of nitric acid overnight at 95 °C. Samples were cooled to room temperature and diluted to a final volume 100 × the weight of the initial sample. Cd, Zn, Cu and all other metals are reported on a µg/g tissue wet weight basis.

### 2.8. Statistical Analysis

All data were analyzed using the GraphPad Prism program (v. 9.0.0, San Diego, CA, USA). For all data sets containing different treatment groups at different time points, a two-way ANOVA was performed followed by Bonferonni post-tests. For comparisons of fat pad weight between control and Cd-treated animals a *t*-test was performed. A *p*-value of ≤0.05 was considered statistically significant.

## 3. Results

### 3.1. Cadmium Accumulation in Renal Cortex

All animals tolerated the Cd injections very well with none being withdrawn due to health concerns. Blood and urine levels of Cd are routinely used to monitor and assess Cd exposure in humans. However, blood and urine samples only indicate recent exposures to Cd [26]. Therefore, the Cd content of the renal cortex was measured because the renal cortex is considered the greatest deposition site of Cd and the most accurate measure of long-term exposure, see Table 1.

Of note is that the Cd content in the renal cortex is roughly the same reported in humans following long-term non-occupational exposure to Cd [2,3].

### 3.2. Cd and Oral Glucose Tolerance Tests

**Figure 1:**
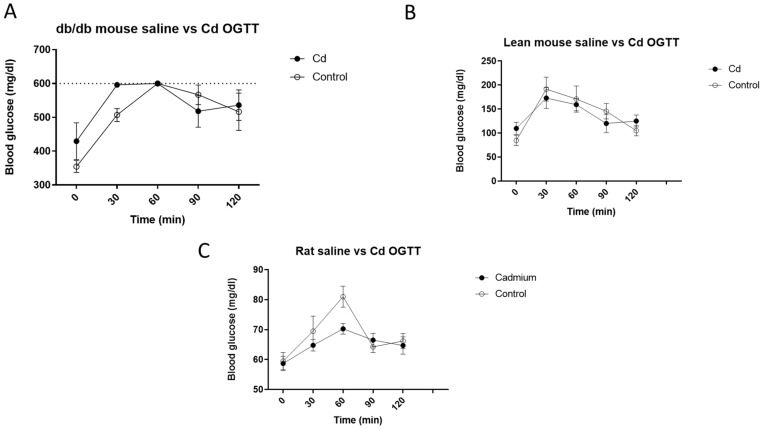
OGTT data following two weeks of 0.6 mg/kg/b.w./day of Cd dosing and two subsequent weeks of no Cd dosing for db/db mice (**A**), lean mice (**B**) and rats (**C**). No statistically significant changes were detected; however, all but one db/db mouse administered Cd had blood glucose levels that exceeded the 600 mg/dL at the 30 min time point, the upper limit of the measuring range of the vsglucose meter (dotted line). Two-way ANOVA with Tukey’s multiple comparison post-tests test; *p* ≤ 0.05 was considered significant; N = 4 per group for mice and N = 6 for rats per group; data are mean ± SE.

To examine the effects of Cd on blood glucose, we dosed animals for two weeks at 0.6 mg/kg/b.w./day, then no further Cd was given for two weeks before we conducted the OGTT. All animals underwent the OGTT without incident. Figure 1A shows the effects of Cd on blood glucose levels in db/db mice (A), lean mice (B) and rats (C) following an oral dose of glucose (2 g/kg); each data point is mean ± SE, N = 4 per group for mice and N = 6 for rats. Surprisingly, at the 30 min time point after glucose administration for the db/db mice, blood glucose levels exceeded the measurable limits of the glucose meter which was 600 mg/dL in three of the Cd-treated db/db mice with the other individual having a value of 584 mg/dL. None of the control db/db mice had blood glucose levels ≥ 600 mg/dL at the 30 min time point. At 60 min, all Cd- and non-Cd-treated db/db mice had blood glucose levels at or above 600 mg/dL. In both the lean mice (Figure 1B) and rats (Figure 1C), there was no significant Cd effect on blood glucose levels during the OGTT. Since db/db mice are both insulin-resistant and have impaired insulin release, it is impossible to differentiate if Cd has an effect on impaired insulin release and/or insulin sensitivity based on these data.

### 3.3. Cd Effects on Mediators of Metabolism Found in Blood

**Figure 2:**
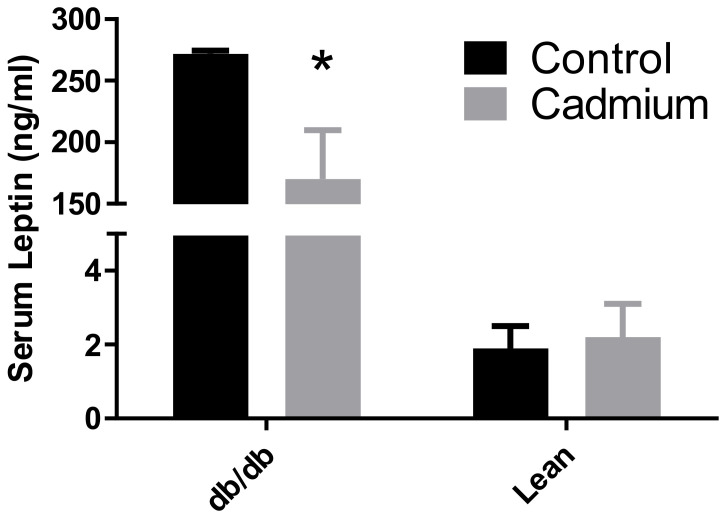
Serum leptin levels following two weeks of 0.6 mg/kg/b.w./day of Cd dosing and two subsequent weeks of no Cd dosing. Asterisk (*) indicates significant differences between saline control and Cd-treated db/db mice. Two-way ANOVA with Tukey’s multiple comparison post-tests test; *p* ≤ 0.05 was considered significant; N = 4 per group for mice and N = 6 for rats per group; data are mean ± SE.

Serum samples (50–100 µL) were collected from 5 h fasted animals and at least 48 h after OGTT. No significant differences were detected in serum insulin values for any of the groups (data not shown); nor was there any apparent trend in the mean values for insulin among the treatment groups. However, the Cd-exposed db/db mouse group had a significant decrease in serum leptin levels (see Figure 2).

Like insulin, levels of serum glucose-dependent insulinotropic polypeptide (GIP) were found to not be statistically different. However, there were consistent differences in mean GIP values between treatment groups with db/db mice having 20.2 ± 4 and 11.9 ± 5 ng/mL ± SE for control and Cd-treated animals, respectively. The lean mice had GIP values of 4.8 ± 1.6 and 2.1 ± 0.05 ng/mL for control and Cd-treated animals, respectively. Rat GIP values were 0.19 ± 0.08 and 0.07 ± 0.002 ng/mL for control and Cd-treated animals, respectively. Had the standard error values been lower with N values per treatment group higher than 4 for mice and 6 for rats, it is possible that statistically significant differences would have been detected.

### 3.4. Cd and Pancreatic Islet Morphology

Morphometric analyses were performed on H&E-stained pancreatic tissue sections. No statistically significant differences were found for the following islet parameters: area, diameter (minimum), diameter (maximum), diameter (mean), perimeter, roundness, cell count or cell number per islet surface area.

### 3.5. Cd Post-Exposure Effects on Individual Bodyweight Changes over Time

**Figure 3:**
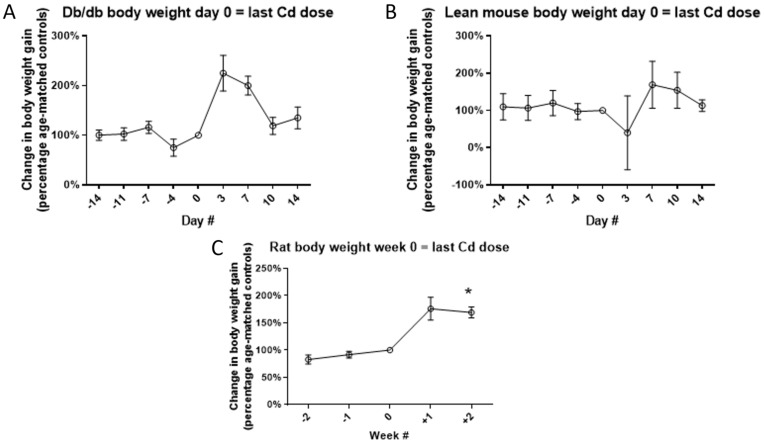
Changes in individual body weight during two weeks of 0.6 mg/kg/b.w./day of Cd dosing and two subsequent weeks of no Cd dosing for db/db mice (**A**), lean mice (**B**) and rats (**C**). Data are percentage of weight gain in Cd-treated animals relative to age-matched saline-treated control animals. Time point “0” is when the last Cd dose was administered. Asterisk (*) indicates significant differences between Cd-treated and week-matched control animals using non-percentage weekly weight gain data. When grouped by control vs. Cd-treatment, the db/db mice (**A**) showed significant differences; however, the multiple comparisons post hoc tests did not show significant differences at any specific time point. Two-way ANOVA with Tukey’s multiple comparison post-tests test; *p* ≤ 0.05 was considered significant; N = 4 per group for mice and N = 6 for rats per group; data are mean ± SE. Note that the deviation in body weight only occurred after Cd dosing had stopped or at time point “0”.

While the average bodyweight was slightly higher in the Cd-treatment group there were no statistically significant differences compared to control animals. It was realized that there is a high level of variation between animals within the same treatment group. Therefore, changes in individual bodyweight were examined and averaged over time, see Figure 3.

### 3.6. Cd Effects on Epidydimal and Retroperitoneal Fat Pad Weight

**Figure 4:**
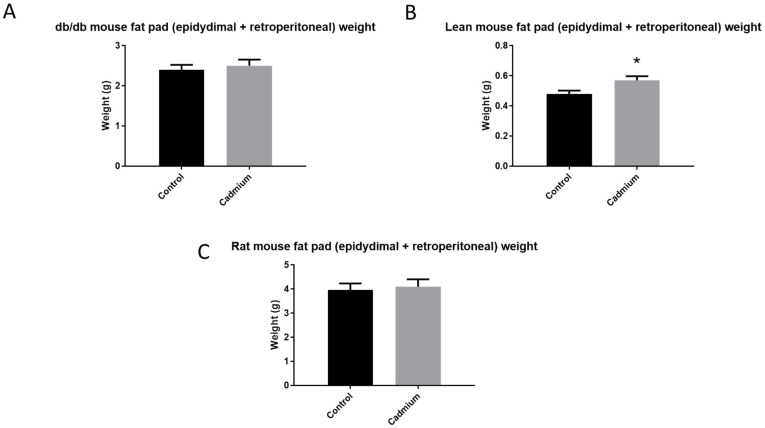
Changes in fat pad weights (epidydimal and retroperitoneal) after two weeks of 0.6 mg/kg/b.w./day of Cd dosing and two subsequent weeks of no Cd dosing for db/db mice (**A**), lean mice (**B**) and rats (**C**). Time point “0” is when last Cd dose was administered. Asterisk (*) indicates significant differences between Cd-treated and control animals. Two-way ANOVA with Tukey’s multiple comparison post-tests test; *p* ≤ 0.05 was considered significant; N = 4 per group for mice and N = 6 for rats per group; data are mean ± SE.

Since bodyweight was showing a divergent trend in Cd-treated animals, indicators of obesity (i.e., fat pad weight) were recorded at the end of the study, see Figure 4. Of note was that the db/db mice had extensive fat accumulation, and therefore, it was difficult to accurately measure fat pad weight for those animals.

## 4. Discussion

This study demonstrated that Cd dosed at 0.6 mg/kg/b.w./day (5 days per week, subcutaneous injection) for two weeks followed by two weeks of no additional Cd exposure in mice and rats, resulted in Cd levels in the renal cortex (Table 1) of similar levels found in the renal cortex of individuals residing in the United States following decades of low-level exposure [2]. This would indicate that this Cd dosing model represents the health effects resulting from long-term Cd accumulation at clinically relevant levels of exposure. Within the renal cortex, several changes in the levels of metals, other than Cd, were found. When all mouse and rat data were pooled together, zinc was higher in the Cd-treated animals (Table 1). This is in agreement with a previously published report from the corresponding author’s lab using the exact same Cd dosing regimen in rats for longer time points such as 6, 9 or 12 weeks [27]. However, in the Prozialeck et al.’s 2016 report, zinc decreased in the later time points of 9 and 12 weeks. This is likely due to the direct nephrotoxic actions of Cd at these later time points causing proximal tubule epithelial cell damage or cell death resulting in the shedding of cellular debris, including zinc, into the glomerular filtrate. Interestingly in rats, copper content was nearly threefold higher but there was no similar change in db/db or lean mice in this study (Table 1). Prozialeck et al., 2016 reported a similar threefold increase in copper for all later 6-, 9- and 12-week time points. This indicates that the robust increase in copper in the renal cortex is specific to rats, at least compared to mice. Additionally, the increase continues after cessation of Cd exposure, and copper levels remain elevated even during proximal tubule damage and shedding into the urinary filtrate. Other studies have shown Cd exposure to have a direct impact on copper concentrations in various tissues including muscle, liver and kidney in sheep and cattle; for a review, see [28].

In addition to altering the metal homeostasis in the renal cortex, this study indicated that Cd may have altered db/db mouse OGTT responses (Figure 1). Although no statistically significant differences were detected by two-way ANOVA, it is possible that there were real effects. At the 30 min time point in the OGTT, all but one Cd-treated db/db mouse had blood glucose levels exceeding the upper limit of the measuring range of the glucose meter which was 600 mg/dL. In addition, the number of animals was low at N = 4 for mice in each treatment group. This was a pilot study, and as such, there were limited resources with unanticipated results. It is informative that only db/db mice, which have reduced leptin receptor activity, were the most impacted by Cd during the OGTT. Leptin is primarily produced in white adipose tissue with a direct correlation between blood leptin levels and amounts of adipose tissue and controls a variety of biological and behavioral aspects including satiety [29]. Plasma leptin levels decrease after fasting but increase drastically after a meal [30]. Leptin is released into the blood from preformed vesicles in a calcium-dependent manner, with insulin being an important enhancer of leptin release but not leptin synthesis in adipocytes [31]. Similar to the current findings in Figure 2, Cd has been reported to cause a decrease in plasma leptin levels in fasted normal and metallothionein-null mice [32]. Furthermore, in vitro studies have shown leptin release to be impaired from human trophoblast cells after exposure to Cd [33]. It is unclear what function Cd may have on adipocytes; however, inflammation and resulting immune system responses have been proposed [32]. In humans, the changes in plasma leptin levels associated with cessation of smoking or reduced Cd exposure appear to be transitory. Months after smoking cessation, leptin levels increase significantly then decrease to previous levels months later [34]. Using the same rat model of Cd exposure as the current study with an extended duration of exposure (3 months of continuous dosing) resulted in decreased plasma leptin levels [35]. Again, it is unknown how Cd causes the decrease in blood leptin levels in these experimental models, or how this effect may impact blood glucose levels.

One surprising result from the current study was the effect Cd had on body weight (Figure 3). Cadmium has long been reported to decrease body weight gain or reduce mean body weight values in a dose- and time-dependent manner in experimental studies using rodents [16,17,36,37]. In adult ovariectomized cynomolgus monkeys given Cd intravenously via the tail vein at doses of either 1.0 or 2.5 mg/kg three times a week for over a year, decreased bodyweight was observed at the highest Cd dose beginning after six months of treatment [36]. Interestingly, it was noted that there was no change in appetite or food consumption in any of the monkeys. Similar Cd effects on body weight are reported in humans. Using NHANES 99-02 data Cd, as measured in urine samples, was one of several metals (lead, cobalt and cesium) found to be negatively correlated with BMI and waist circumference in both adults and adolescents (ages 6–18) [37]. Cigarette smoke is the primary source of non-occupational Cd exposure [19]. Studies examining cigarette smoking and obesity found smoking to be negatively correlated with BMI [38]. However, the same study found ex-smokers to be at increased risk for obesity as measured by BMI. The interesting and novel finding reported here is that low dose Cd has a greater effect on increasing body weight gain following cessation of exposure (last two weeks) than reducing body weight during the exposure period (first two weeks).

The effects of Cd on white adipose tissue reported here (Figure 4) are also of interest. In an experimental study using very similar Cd doses (0.5 and 0.75 mg/kg/b.w./day, s.c.) and duration of exposure (7 days), one week after the last dose of Cd was administered, there was no change in body weight or white adipose tissue in normal (metallothionein +/+) mice [32]. However, in the same study, metallothionein-null mice showed a decrease in body weight and decreased white adipose tissue. In these metallothionein-null animals, the morphology of adipose tissue changed with smaller sized individual adipocytes [32]. We report here that, at two weeks following the last Cd dose, white adipose tissue (epidydimal and retroperitoneal) weight was significantly increased in the Cd group for normal or lean mice. It may be likely that had the Kawakami et al., 2013 study sampled adipose tissue a week later, significant increases in adipose tissue weight could have occurred in the normal (metallothionein +/+) mice. It is unclear why the metallothionein-null mice would have shown decreases in body weight and white adipose tissue one week after the last Cd dose was administered.

There were several shortcomings in this study, including the aforementioned low N value for mice and blood glucose levels exceeding the upper limit of the measuring range. In addition, serum was used as a sample to determine values of mediators of metabolism—insulin, GIP and leptin. Plasma is preferred over serum due to a higher stability resulting from less enzymatic degradation of proteo-hormones, i.e., leptin and insulin [39]. Additionally, considering the importance of leptin in controlling appetite, we did not record individual food intake during or following Cd exposure. Lastly, in any rodent model of diabetes, it is important to acknowledge that the etiology of diabetes mellitus spans years if not decades of time. Examining the long-term effects of diabetes is not possible in a rodent with a life span of two years. Therefore, we can only extrapolate.

Overall, this study shows that Cd exposure at a level that mirrors life-long human exposure results in diabetogenic as well as obesogenic effects. Further study is needed to identify how Cd may alter leptin levels, which may adversely affect blood glucose levels. Of great interest is to determine how Cd alters bodyweight and specifically white adipose tissue weeks after the last Cd dose is administered. This research could have implications in global efforts to reduce tobacco use considering the importance of cigarettes as a source of Cd exposure and the profound effects cigarette smoke has on body weight. How important the exposure to environmental substances is in determining the likelihood of a person becoming diabetic or obese remains to be determined. However, studies such as this would indicate that exposure to environmental toxicants such as Cd certainly warrant further investigation.

## Figures and Tables

**Table 1:** Levels of metals in renal cortex (µg/g wet weight) after 2 weeks of 0.6 mg/kg/b.w./day of Cd dosing and 2 subsequent weeks of no Cd dosing; control animals received equal volume vehicle (saline) injections. Asterisk (*) indicate significant differences between control vs. cadmium treatment groups for whole data set; ^a^ = significant differences compared to Cd treatment within the same species/strain. All data are expressed as mean ± standard error µg/g wet weight. Two-way ANOVA with Tukey’s multiple comparison post-tests test; *p* ≤ 0.05; n = 4 per group for mice and n = 6 for rats per group.

	Boron	Cadmium	Calcium	Copper	Iron	Magnesium	Manganese	Molybdenum	Phosphorus	Potassium	Sodium	Zinc *
Control lean mouse	12.8 ± 6.5	^a^ 0.82 ± 0.33	121 ± 44	5.62 ± 1.0	81.5 ± 10	255 ± 19	2.02 ± 0.24	0.88 ± 0.3	4373 ± 279	5271 ± 433	1290 ± 150	41.0 ± 5.9
Cadmium lean mouse	7.73 ± 1.1	45.4 ± 3.6	91.1 ± 3.0	5.72 ± 0.52	74.2 ± 7.6	260 ± 13	2.14 ± 0.05	0.68 ± 0.04	4338 ± 239	5246 ± 411	1305 ± 143	45.0 ± 2.6
Control db/db mouse	4.28 ± 0.61	^a^ 0.4 ± 0.04	81.2 ± 2.7	5.1 ± 0.17	68.2 ± 5.6	243 ± 8.4	1.81 ± 0.03	0.74 ± 0.02	3938 ± 100	4797 ± 277	1080 ± 111	32 ± 0.41
Cadmium db/db mouse	4.88 ± 1.0	46.3 ± 5.7	91.9 ± 16	5.87 ± 0.43	82.2 ± 7.5	246 ± 7.1	1.96 ± 0.08	0.80 ± 0.07	3983 ± 125	4944 ± 385	1157 ± 140	46.7 ± 4.8
Control rat	2.22 ± 0.38	^a^ 0.26 ± 0.07	89.5 ± 4.8	^a^ 7.42 ± 0.21	51.6 ± 4.4	212 ± 5.8	0.95 ± 0.03	0.38 ± 0.05	3281 ± 83	3691 ± 172	1026 ± 38	31.1 ± 2.1
Cadmium rat	2.59 ± 0.66	71.24 ± 2.9	83.7 ± 7.3	22.8 ± 1.8	53.6 ± 7.2	194 ± 3.7	0.92 ± 0.02	0.52 ± 0.12	3059 ± 57	3425 ± 89	1100 ± 58	42.3 ± 2.3

## Data Availability

The data presented in this study are available upon request from the corresponding author.
